# Vincristine dosing, drug exposure and therapeutic drug monitoring in neonate and infant cancer patients

**DOI:** 10.1016/j.ejca.2021.09.014

**Published:** 2022-03

**Authors:** Shelby Barnett, Farina Hellmann, Elizabeth Parke, Guy Makin, Deborah A. Tweddle, Caroline Osborne, Georg Hempel, Gareth J. Veal

**Affiliations:** a Newcastle University Centre for Cancer, Newcastle University, Newcastle upon Tyne, UK; b Department of Pharmaceutical and Medical Chemistry, University of Münster, Münster, Germany; c Division of Cancer Sciences, University of Manchester, Manchester, UK; d Royal Manchester Children's Hospital, Manchester, UK; e Great North Children's Hospital, Newcastle, UK; f Pharmacy Department, Alder Hey Children's NHS Foundation Trust, Liverpool, UK

**Keywords:** Vincristine, Chemotherapy, Pharmacokinetics, Dosing, Paediatrics, Neonates, Infants, Therapeutic drug monitoring

## Abstract

**Background:**

The anticancer drug vincristine is associated with potentially dose-limiting side-effects, including neurotoxicity and myelosuppression. However, there currently exists a lack of published clinical pharmacology data relating to its use in neonate and infant patients. We report a study investigating vincristine dosing and drug exposure, alongside the feasibility and impact of a therapeutic drug monitoring treatment approach, in this challenging patient population.

**Patients and methods:**

Vincristine pharmacokinetic data from a total of 57 childhood cancer patients, including 26 neonates and infants, were used to characterise a population pharmacokinetic model. Vincristine was administered at doses of 0.02–0.05 mg/kg or 0.75–1.5 mg/m^2^ in neonates and infants aged <1 year or ≤12 kg and doses of 1.5 mg/m^2^ in older children.

**Results:**

A two-compartment model provided the best fit for the population analysis. There was no significant difference in vincristine clearance normalised for body surface area between neonates/infants and older children. Lower doses administered to neonates and infants resulted in significantly lower drug exposures (area under the curve [AUC]), compared with older children (p = 0.047). Vincristine doses of <0.05 mg/kg in neonates and infants resulted in significantly lower AUC values than observed in those receiving doses of ≥0.05 mg/kg (p ≤ 0.0001). Therapeutic drug monitoring was shown to be feasible, effective and well tolerated in neonates and infants experiencing suboptimal drug exposures.

**Conclusion:**

Doses of <0.05 mg/kg should not be used in neonate and infant patients because of a high risk of patients experiencing potentially suboptimal drug exposures. Therapeutic drug monitoring approaches in neonates and infants are supported by the data generated, with a proposed target therapeutic window of 50–100 μg/l∗h.

## Highlights

• Vincristine dosing and drug exposure was investigated in neonates and infants.
• Vincristine concentrations were quantified in 210 plasma samples from 57 children.
• Lower drug exposures were observed in infants and neonates compared with older children.
• Therapeutic drug monitoring can be used to avoid suboptimal vincristine drug exposures.
• Vincristine dosing guidance is provided for treatment of neonate and infant patients.

## 1 Introduction

Similar to many well-established anticancer drugs, the use of the tubulin-binding agent vincristine is associated with potentially dose-limiting side-effects, including neurotoxicity and myelosuppression [1,2]. These adverse effects can severely impact quality of life and may also lead to treatment delays or even discontinuation of vincristine treatment [3]. The administration of anticancer drugs with narrow therapeutic windows, such as vincristine, represents a particular clinical challenge in the treatment of infants and neonates. Although it is imperative that dosing regimens used provide the best chance of eliciting a clinical response, it is equally important to consider the potential short- and long-term effects of treatment in a clearly vulnerable patient population. This is particularly important for a drug such as vincristine, which is widely used across a range of paediatric malignancies, including leukaemias, lymphomas, sarcomas, brain tumours and neuroblastoma [4,5]. Indeed, a recent report from the Children's Oncology Chemotherapy Standardization Task Force highlighted the lack of standardisation in vincristine dosing for infants/neonates, both in the application of dose modifications and in the range of milestones used for infant classification [6].

Developmental physiological changes occurring in the first weeks and months after birth can have a considerable impact on drug disposition, with marked variability in drug exposure observed in neonates and infants for a number of anticancer drugs where clinical pharmacology studies have been conducted [[7], [8], [9]]. The widely accepted approach to the dosing of chemotherapeutics such as vincristine is to either shift from surface area–based dosing (mg/m^2^) to body weight–based dosing (mg/kg) or to implement 20–50% dose reductions in children aged <1 year or below a defined body weight (commonly 10 or 12 kg) compared with older children. However, there would appear to be a lack of published clinical pharmacology data underpinning these dosing regimens [[10], [11], [12]]. With a fine balance existing between the achievement of a desirable clinical response and the avoidance of the more severe adverse effects of vincristine, it would seem advantageous to further develop our knowledge of the clinical pharmacology of this widely used anticancer drug in a clearly important yet susceptible patient population. Such information may support the utility of routine therapeutic drug monitoring dosing for vincristine, an approach that has previously been shown to be advantageous for the treatment of neonates and infants with the anticancer drug carboplatin [13,14].

In the present study, we investigate the clinical pharmacology of vincristine in neonate and infant patients compared with older children, in terms of the dosing regimens commonly used and differences in pharmacokinetics and drug exposures observed across the paediatric age spectrum. The feasibility and impact of adaptive drug dosing were also investigated in patients experiencing low vincristine exposures. Data generated have the potential to directly impact dosing regimens taken forward in neonate and infant patient populations.

## 2 Materials and methods

### 2.1 Patients and treatment

The study included data obtained from three separate national multicentre clinical pharmacology studies (ISRCTN studies ISCRTN52616678, ISRCTN64515327, and ISCRTN10139334). All study protocols were approved by the local UK Research Ethics Committee, and written informed consent was obtained from all patients or parents, as appropriate. Eligible patients were those aged < 21 years who were receiving vincristine as part of their standard chemotherapy for a range of cancer types. To participate in the study, patients were required to have a central venous catheter in place to facilitate the collection of sequential blood samples for pharmacokinetic analysis and a glomerular filtration rate of >60 mL/min/1.73 m^2^. Patient characteristics including age, gender, body weight and body surface area were recorded. Baseline toxicity data were collected before and following vincristine administration.

Vincristine was administered as a short bolus infusion at doses of 0.02–0.05 mg/kg or 0.75–1.5 mg/m^2^ in neonates and infants aged <1 year or ≤12 kg and doses of 1.5 mg/m^2^ (with dose capping at 2 mg) in older children or as part of their standard treatment regimen. Toxicity was assessed after treatment on the cycle that pharmacokinetic samples were collected by the National Cancer Institute Common Terminology Criteria of Adverse Events, version 4.0.

### 2.2 Blood sampling and analysis

Blood samples were collected from the patient's central line at multiple time points after drug administration, commonly at 0.25, 0.5, 1, 2, 4, 6, 8 and 24 h, although more limited sampling was carried out for some patients. Drug administration and sampling times were accurately recorded, and all samples were collected from a different lumen to that used for drug administration following a standardised procedure. After collection, blood samples (1–3 mL) were immediately centrifuged (1200*g*, 4°C, 10 min), and the plasma obtained was stored at −20°C. Plasma samples were transported by overnight courier on dry ice in an insulated container to the Newcastle University Centre for Cancer for analysis. Quantification of vincristine levels in plasma samples was carried out using a validated liquid chromatography–mass spectrometry assay, with a lower limit of quantification (LLOQ) of 0.50 ng/mL, as previously described [15,16]. Intra-assay coefficients of variation were <10% in all cases.

### 2.3 Population pharmacokinetic analysis

Drug concentration data were used to generate a population pharmacokinetic (pop PK) model for vincristine. The pop PK analysis was performed using NONMEM (version 7.3; Icon Development Solutions, Ellicott City, MD, USA), Perl speaks NONMEM 4.9.0, Pirana (version 2.9.7), R 4.0.5 and RStudio (version 1.4.1106). Pharmacokinetic parameters and their variability were estimated using the first-order conditional estimation method with interaction. Vincristine plasma concentrations were log transformed for model development. Interindividual variability was tested on all pharmacokinetic parameters with exponential models. Residual variability was described with a log error model. To account for a potential impact of size in this paediatric population, allometric scaling (Eq. (1)) as well as body surface area scaling (Eq. (2)) were tested on all pharmacokinetic parameters.

(1) $$\text{TVP}={\text{θ}}_{\text{P}}*{\left(\frac{\text{BW}}{{\text{BW}}_{\text{median}}}\right)}^{\text{k}}$$

(2) $$\text{TVP}={\text{θ}}_{\text{P}}*(1+{\left(\frac{\text{BSA}}{{\text{BSA}}_{\text{median}}}\right)}^{\text{θ}}$$

In Eqs. (1), (2), TVP represents the typical value of the pharmacokinetic parameter, and θ_P_ represents the population value of the pharmacokinetic parameter. In Eq. (1), the exponent K is fixed to 0.75 for clearances and fixed to 1 for volumes of distribution. The exponent θ in Eq. (2) is separately estimated for clearances and for volumes of distributions by the model. Covariates of interest were selected based on graphical assessment and thereafter tested using a stepwise covariate model (SCM) building approach. In the forward inclusion step, a reduction of the objective function value (OFV) of 3.84 (p < 0.05) for 1 degree of freedom was sufficient to retain the covariate in the model. In the backward elimination step, a covariate was removed from the model if the OFV increased by <6.64 (p < 0.01) for 1 degree of freedom. For categorical covariates, a linear relationship was evaluated, with linear, piecewise linear and exponential relationships tested for continuous covariates. Alongside comparison of OFV for nested models, the Bayesian information criterion (BIC) calculated by Pirana was used to compare non-nested models. Models with a lower BIC were considered superior.

### 2.4 Model evaluation

For model evaluation, goodness-of-fit (GOF) plots and visual predictive checks (VPC) were generated, and a bootstrap was performed. GOF plots include the following scatterplots: (1) observed versus individual predicted concentrations (DV versus IPRED), (2) observed versus population-predicted concentrations (DV versus PRED), (3) conditional weighted residuals versus population-predicted concentrations (CWRES versus PRED) and (4) conditional weighted residuals versus time (CWRES versus time). For the VPC, a simulation of 500 data sets based on the model was performed in NONMEM. The 5%, 50% and 95% percentiles of the observed concentrations were plotted in combination with the medians of the 5%, 50% and 95% percentiles and their 95% confidence interval (CI) of the simulated concentrations versus time. The graphical output was generated using the tidyvpc package in R 4.0.5. The bootstrap was conducted using 1000 replicates of the original data set. Based on the bootstrap data, the median and the 95% CI of each model parameter were determined.

### 2.5 Post-hoc parameter analysis

In the post-hoc parameter analysis, individual exposure metrics parameters and the individual clearance (CL) values were analysed. Area under the curve (AUC), maximal plasma concentration (C_max_) and time of C_max_ (T_max_) were estimated using the final pop PK model. For the graphical assessment of potential relationships between the individual post-hoc parameters and covariates of interest, scatterplots were generated.

### 2.6 Therapeutic drug monitoring

After initial dosing according to the relevant treatment protocol on cycle 1, vincristine doses on subsequent cycles were increased at the discretion of the treating clinician, if vincristine exposure was potentially suboptimal and/or if it was felt that a better clinical response could be obtained at a higher dose. Dosing decisions were based on an assessment of drug exposure on cycle 1, alongside clinical response and toxicity data. Drug exposures were measured on multiple cycles in some patients at the request of the treating clinician.

### 2.7 Statistical analysis

Statistical analysis was performed using non-parametric tests, including Spearmen rank correlation and Wilcoxon rank-sum test or parametric tests including one-way analysis of variance and Welch's t-test, as appropriate, using R 4.0.5 and RStudio (version 1.4.1106) or GraphPad Prism version 9.0 (GraphPad Software, La Jolla, CA, USA). A significance level of p < 0.05 was assumed.

## 3 Results

### 3.1 Patient characteristics and treatment

A total of 57 patients receiving vincristine as part of their standard chemotherapy were recruited onto the studies between October 2014 and March 2021. The study population had a median age of 5.6 years (range: 2 weeks–17.2 years) and included a total of 26 neonate and infant patients (aged <1 year and/or <12 kg). The neonate and infant patients were receiving treatment for or a range of solid tumours, including rhabdomyosarcoma, Wilms tumour and glioma, whereas the older patients were being treated for acute lymphoblastic leukaemia (ALL). A summary of patient characteristics is provided in Table 1. Doses of vincristine were dependent on tumour type and treatment protocol and ranged from 0.02 mg/kg (equivalent to 0.35 mg/m^2^) to 1.5 mg/m^2^.

**Table 1:** Patient characteristics and treatment.

Characteristic	No.
Evaluable patients	57
**Age (months)**	
0–1	3
2–12	18
13–24	5
>24	31
**Sex**	
Male	36
Female	21
**BW (kg)**	
Median	19.8
Range	2.9–84.7
**BSA (m**^**2**^**)**	
Median	0.78
Range	0.21–2.1
**Tumour type**	
ALL	31
Rhabdomyosarcoma	6
Wilms tumour	5
Glioma	4
Ewings sarcoma	2
Rhabdoid tumour	2
Neuroblastoma	1
Astrocytoma	1
Ependymoma	1
Burkitt's lymphoma	1
Peripulmonary blastoma	1
Brain tumour	1
Infantile fibrosarcoma	1

### 3.2 Vincristine pharmacokinetics

In total, 57 patients with 210 plasma concentrations were available in the vincristine data set. Eleven plasma concentrations were below the lower limit of quantification (LLOQ = 0.5 ng/mL) and were excluded from the pop PK analysis. Therefore, the vincristine pop PK analysis was conducted based on 57 patients and 199 plasma concentrations.

A two-compartment model with first-order elimination was developed. Allometric scaling was implemented on all pharmacokinetic parameters, as it resulted in a lower OFV of −58.5 compared with body surface area scaling (OFV = 50.3). Interindividual variability was evaluated on CL, intercompartmental clearance (Q2) and peripheral volume of distribution (V2). The residual variability was described using a log error model. Age and study were regarded as covariates of interest. In the SCM, both covariates were identified as having a significant effect on V2. The age effect was included using a linear relationship. For study, a linear effect was added for neonate and infant patients, leading to a lower V2. The inclusion of both covariates resulted in a decrease of 15.2 in OFV and a decrease of 10.4% of interindividual variability of V2. Overall, the precision of the model parameters was good, with relative standard errors (RSEs) lower than 22%. The final model parameter estimates are presented in Table 2.

**Table 2:** Parameter estimates of final vincristine model and bootstrap results.

Parameter	Final model			Bootstrap	
	Estimates	RSE (%)	Shrinkage (%)	Median	95% CI
CL (L/h)^a^	13.4	8		13.4	11.4 to 16.0
V1 (L)^a^	8.31	17		8.31	5.64 to 12.71
Q2 (L/h)^a^	77.6	17		77.5	56.1 to 115.9
V2 (L)^a^	303	18		296	181 to 406
Log error (%)	23.1	12	33	22.7	17.5 to 28.9
Study on V2	−0.638	12		−0.620	−0.749 to −0.297
Age on V2	−0.0537	19		−0.0521	−0.0684 to −0.0049
IIV on CL (%)	36.5	21	31	35.3	18.7 to 72.6
IIV on Q2 (%)	77	11	15	73.9	52.0 to 97.7
IIV on V2 (%)	43.9	15	14	41.9	18.3 to 56.6

a Allometric scaling was applied to all PK parameters. The parameters are given for a patient with a body weight of 19.8 kg.

### 3.3 Model evaluation

**Fig. 1:**
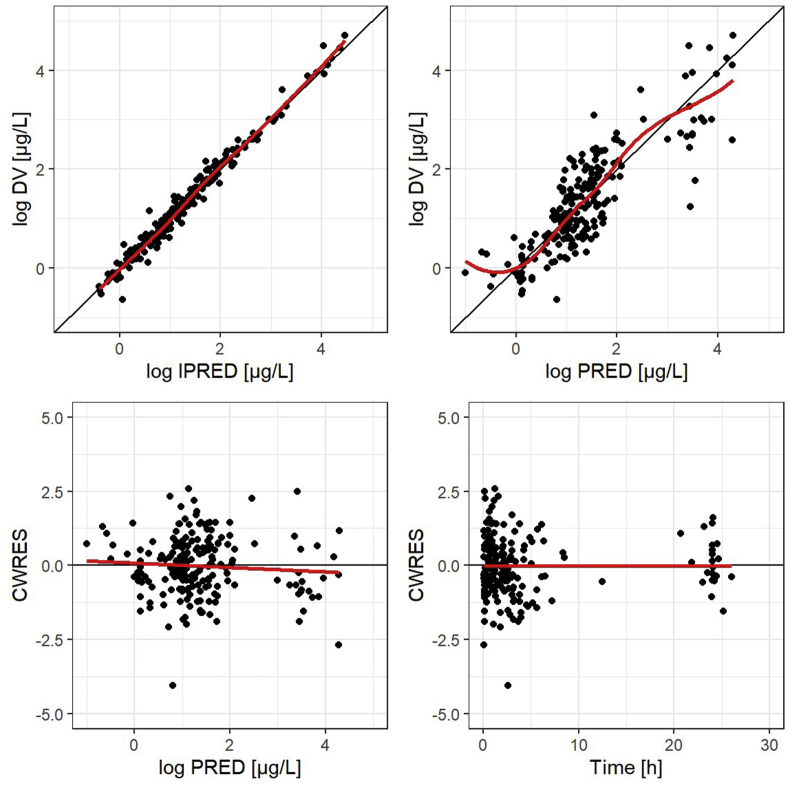
Goodness-of-fit plots for the final vincristine model. Plasma concentrations for DV, IPRED and PRED are log transformed. CWRES, conditional weighted residuals; DV, observed plasma concentrations; IPRED, individual predicted plasma concentrations; PRED, population-predicted plasma concentrations.

**Fig. 2:**
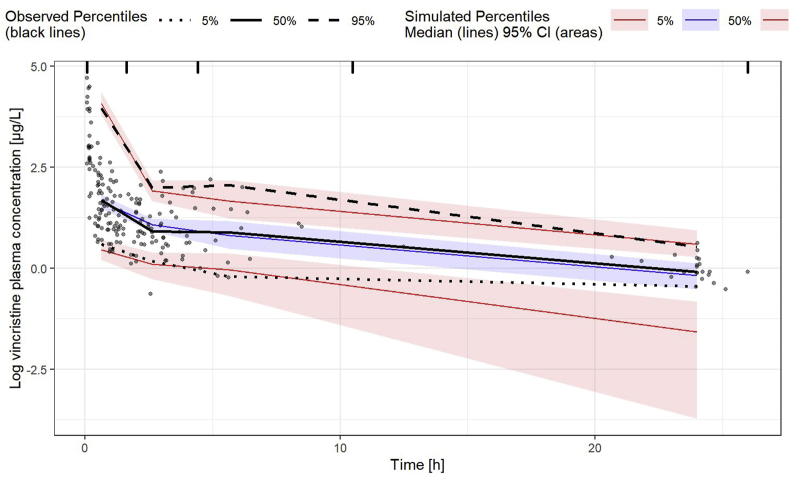
Visual predictive check (VPC) of final vincristine model. Plasma concentrations are log transformed. CI, confidence interval.

The GOF plots indicate a good model fit of the final pop PK model for vincristine as shown in Fig. 1. Besides the final parameter estimates, Table 2 exhibits the results of the performed bootstrap with 1000 replicates. The convergence rate of the bootstrap is 96.5%. The parameter medians of the bootstrap are close to the final model parameter estimates. Furthermore, the 95% CIs of the bootstrap parameters contain the parameter estimates of the final vincristine pop PK model. The results of the bootstrap indicate a good precision of the final model parameter estimates and an adequate model stability. Fig. 2 presents the VPC of the final pop PK model for vincristine. The VPC illustrates that the model describes the data adequately. The 5th, 50th and 95th percentiles of the observed plasma concentrations are contained in the 95% CI of the 5th, 50th and 95th percentiles of the simulated plasma concentrations. The 5th percentile of the observed plasma concentrations represented by the black dotted line lies slightly above the 95% CI of the 5th percentile of the simulated plasma concentrations. This might indicate an underprediction of late plasma concentrations and represents a potential limitation of the model. However, for those late samples taken later than 15 h after the start of vincristine administration, only a few observed plasma concentrations were available. The availability of late plasma concentration data was particularly an issue for neonate and infant patients in the present study population.

### 3.4 Vincristine pharmacokinetics and age

**Fig. 3:**
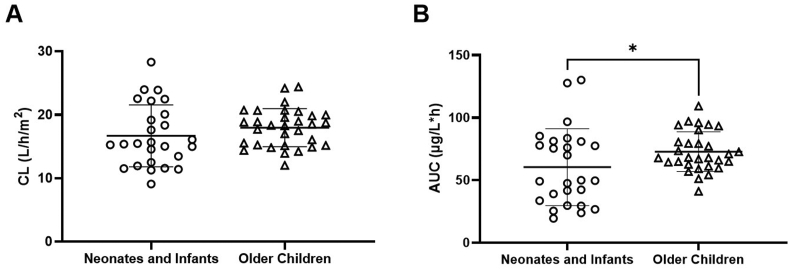
Comparison of (A) vincristine clearance normalised to body surface area and (B) vincristine AUC in neonates and infants (n = 26) compared with older children (n = 30; ∗p < 0.05).

Vincristine pharmacokinetic parameters were determined in neonate and infant patients (<1 year of age or ≤12 kg) receiving 0.02–0.05 mg/kg or 0.75–1.5 mg/m^2^, compared with older children receiving doses of 1.5 mg/m^2^ (with dose capping at 2 mg). Table 3 provides a summary of the key pharmacokinetic parameters in neonates and infants compared with older children. There was no significant difference in CL values normalised for body surface area between neonates/infants and older children as shown in Fig. 3A (p = 0.46). Because of the lower doses administered to the neonate and infant patient population compared with older children, this resulted in significantly lower drug exposures, as determined by observed AUC values, being achieved in the younger patient group as shown in Fig. 3B (p = 0.047). There was a trend toward lower CL values in neonates (0–4 weeks of age) compared with infants (1–12 months of age), with CL values ranging from 9.1 to 15.7 L/h/m^2^ and 11.3 to 28.3 L/h/m^2^, respectively. However, the very limited numbers of patients in the former group did not allow a meaningful statistical analysis to be conducted.

**Table 3:** Vincristine pharmacokinetic parameters observed in neonates and infants (aged <1 year and/or <12 kg) compared with older children.

Parameter	Neonates and infants (N = 26)	Older children (N = 30)	Overall (N = 56)
**AUC (μg/l∗h)**			
Mean (SD)	59.8 (30.9)∗	72.9 (15.9)∗	66.8 (24.7)
Median (Min, Max)	53.6 (17.9, 130)	68.1 (41.0, 110)	67.5 (17.9, 130)
**C**_**max**_**(μg/l)**			
Mean (SD)	88.2 (37.8)	83.7 (18.4)	85.8 (28.8)
Median (Min, Max)	90.9 (17.7, 162)	85.3 (39.5, 126)	85.7 (17.7, 162)
**T**_**max**_**(h)**			
Mean (SD)	0.0692 (0.0934)	0.0580 (0)	0.0632 (0.0632)
Median (Min, Max)	0.0500 (0.0167, 0.500)	0.0580 (0.0580, 0.0580)	0.0580 (0.0167, 0.500)
**CL BSA normalised (L/h/m**^**2**^**)**			
Mean (SD)	17.0 (5.05)	17.9 (3.05)	17.5 (4.09)
Median (Min, Max)	15.4 (9.09, 28.3)	18.3 (12.0, 24.4)	17.6 (9.09, 28.3)
**V1**			
Mean (SD)	2.81 (0.987)	15.7 (6.83)	9.71 (8.19)
Median (Min, Max)	2.79 (1.22, 4.60)	14.2 (7.64, 35.5)	8.08 (1.22, 35.5)

∗Significant difference in AUC values observed between neonates/infants and older children (p < 0.05).

**Fig. 4:**
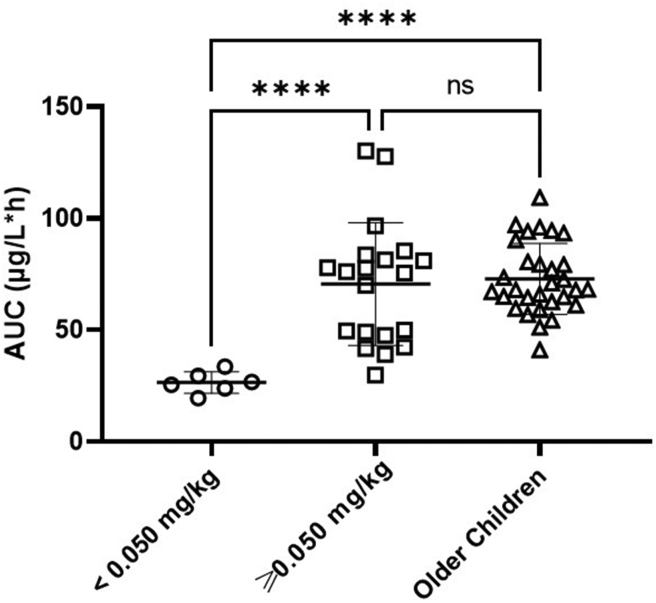
Vincristine exposure (AUC) values observed in neonate and infant patients following doses of <0.05 mg/kg and doses of ≥0.05 mg/kg, compared with exposures observed in older children receiving a dose of 1.5 mg/m^2^ (∗∗∗∗p < 0.0001).

Neonates and infants received a wide range of doses ranging from 0.02 mg/kg (equivalent to 0.35 mg/m^2^) to 1.5 mg/m^2^ depending on treatment protocol. Looking at the observed exposures associated with the doses administered in these younger patients, it can be seen that vincristine doses of <0.05 mg/kg result in significantly lower AUC values than observed in neonates and infants receiving doses of ≥0.05 mg/kg (p < 0.0001) and in older children receiving a dose of 1.5 mg/m^2^ (p < 0.0001) as shown in Fig. 4. There was no significant difference in vincristine exposures between younger patients receiving vincristine doses of ≥0.05 mg/kg and older children. In the older patient population, there was no significant difference observed in drug exposure between those patients who had their vincristine dose capped at 2 mg because of larger body surface areas, compared with those patients where dose capping was not required.

### 3.5 Therapeutic drug monitoring

Vincristine doses were adjusted in seven patients at the discretion of the treating clinician, following initial doses of 0.025 mg/kg in one patient, 0.05 mg/kg in four patients and 1.125 mg/m^2^ in two patients. Vincristine doses were increased to 1.5 mg/m^2^ in all but one case, where the dose was increased from 0.025 mg/kg to 0.05 mg/kg. The dose adjustments implemented on each cycle of treatment and the corresponding C_max_ and AUC values achieved are shown in Table 4. Vincristine exposures in these patients increased by 37–110% with the dose increases implemented, with all patients achieving a drug exposure approaching or above the median AUC value of 68.1 μg/l∗h observed in the older paediatric patient population studied (Table 3), at the increased dose level. For patients where samples for pharmacokinetic analysis were collected on more than one cycle at the same dose level, observed AUC values between cycles were within 10% of each other.

**Table 4:** Vincristine dosing and exposure data across treatment cycles for therapeutic drug monitoring patients.

Pt	Cycle 1				Cycle 2				Cycle 3			
	BW (kg)	Dose	C_max_ (ng/ml)	AUC (μg/l∗h)	BW (kg)	Dose	C_max_ (ng/ml)	AUC (μg/l∗h)	BW (kg)	Dose	C_max_ (ng/ml)	AUC (μg/l∗h)
25	9.6	1.125 mg/m^2^	112	75.4	10.1	1.5 mg/m^2^	165	107				
03	4.4	0.05 mg/kg	89.8	39.0	4.9	1.5 mg/m^2^	163	72.1				
14	7.1	1.125 mg/m^2^	143	49.1	7.3	1.125 mg/m^2^	146	53.9	8.5	1.5 mg/m^2^	187	67.1
20	6.5	0.025 mg/kg	32.4	26.7	7.0	0.05 mg/kg	72.7	56.2				
04	7.9	0.05 mg/kg	110	42.3	8.6	1.5 mg/m^2^	150	64.6	9.9	1.5 mg/m^2^	154	62.6
10	8.8	0.05 mg/kg	60.8	76.1	9.0	1.5 mg/m^2^	83.6	119				
13	3.6	0.05 mg/kg	17.9	49.9	3.6	1.5 mg/m^2^	33.7	94.2				

Vincristine tolerability was investigated in patients where dose increases were implemented based on drug exposure. The increased vincristine dose was well tolerated in five of seven patients, with patients receiving up to 14 additional cycles of treatment at the increased dose level.

For patient 25, the dose increase on cycle 2 was made as the patient crossed the age boundary for receiving full drug dose, despite achieving a good vincristine exposure on cycle 1. The patient experienced haematological toxicity at the higher dose level (1.5 mg/m^2^), and the dose was reduced back to the 75% dose level (1.125 mg/m^2^). This patient therefore had an acceptable drug exposure even at the lower dose, with the dose increase not based on drug exposure. Vincristine levels were measured in patient 10 at an initial dose of 0.05 mg/kg because of poor tolerability at a higher dose on previous cycles. Increasing the dose back up to 1.5 mg/m^2^ in this patient, in an attempt to improve the clinical response, again resulted in unacceptable haematological toxicity, and the dose was decreased again for the remaining cycles of treatment. Importantly, all patients participating in TDM dosing were able to achieve drug exposures equivalent to AUC values observed in the older paediatric patients at the dose level that was well tolerated and taken forward for the remaining cycles (56.1–94.2 μg/l∗h).

## 4 Discussion

There are currently limited published data on cancer drug disposition in neonate and infant cancer patients, resulting in current dosing regimens being based on limited scientific rationale in terms of pharmacological exposure. Indeed, it is remarkable that this remains the case for a drug such as vincristine, which has been a mainstay of treatment for childhood cancer for over 50 years. This is particularly surprising when we consider that evidence from pharmacological studies have positively impacted on how we dose a number of important anticancer drugs in a paediatric setting, including busulfan [17,18], carboplatin [13,14] and most recently, cyclophosphamide [19]. The present study was designed to provide comprehensive data on vincristine pharmacokinetics and drug exposure in neonates and infants, compared with older children, with a view to informing scientifically based dosing guidelines for an understudied and vulnerable patient population. A major goal of the study was to ensure that the patient population covered the full paediatric age spectrum, with a focus on generating comprehensive data in those treated in the first weeks and months of life, a period when drug disposition is most likely to be variable. This was achieved by recruiting a total of 57 patients receiving vincristine as part of their standard clinical treatment, including 26 in the neonate and infant group, with a total of over 200 plasma samples available for pharmacokinetic analysis. In addition, the study investigated the feasibility and potential clinical benefit of using a TDM treatment approach in a subset of neonate and infant patients.

A two-compartment pharmacokinetic model with first-order elimination was developed, with an improved fit obtained by incorporating allometric scaling on all pharmacokinetic parameters. The final model exhibited a good fit and was used to generate estimates of key pharmacokinetic parameters for the study patients. Overall, there was no observed difference in vincristine CL values normalised for surface area between neonates and infants and older children. This finding is consistent with previously published studies investigating vincristine pharmacokinetics in children [[20], [21], [22]]. Because of the lower and more variable doses administered to the neonate and infant patients, ranging from 0.02 to 0.05 mg/kg and 0.75 to 1.5 mg/m^2^, this resulted in significantly lower and more wide-ranging drug exposures being observed compared with the older children, who were dosed consistently at 1.5 mg/m^2^. In particular, vincristine doses of <0.05 mg/kg resulted in significantly lower drug exposures than observed in neonates and infants receiving doses of ≥0.05 mg/kg and in older children receiving a dose of 1.5 mg/m^2^. The six patients receiving vincristine doses of <0.05 mg/kg were aged between 2 and 5 months, with body weights ranging from 4.8 to 9.2 kg and did not include the three neonate patients studied.

These data are consistent with recently published data from a US Children's Oncology Group study, which similarly showed that low vincristine drug exposures were observed in patients receiving doses <0.05 mg/kg [22]. Although a wide range of vincristine AUC values were observed in the neonate and infant patients in our study, from 17.9 to 130 μg/l∗h, this would be reduced to a range of 33.6–130 μg/l∗h if all patients were dosed at ≥0.05 mg/kg, comparable to an AUC range of 41.0–110 μg/l∗h in the older patient population.

In the present study, TDM approaches to treatment were initiated at the request of the treating clinician for seven patients because of concerns regarding the suitability of defined age-based or body weight–based dosing cutoff points recommended in treatment protocols or because of a desire to achieve improved clinical response in patients where response was believed to be suboptimal. The data presented in this subset of patients highlight the feasibility and potential clinical benefits of increasing vincristine doses in patients achieving lower drug exposures, where it is felt that an improved clinical response could be achieved. The dose increases were tolerated well in all patients where exposures were deemed to be below the average for the wider patient population. In further support of using vincristine exposure data to inform dosing, two additional patients who had their doses increased at the request of the treating clinician (patients 10 and 25), despite achieving good exposures at the lower dose level, had to subsequently decrease the dose on additional cycles of treatment because of poor tolerability.

Based on the data generated, we would recommend a target vincristine AUC range of 50–100 μg/l∗h in neonates and infants, exposures that are both achievable and well tolerated, in addition to being comparable to exposures observed in older children in the present study. The use of TDM approaches to ensure that this target therapeutic window is achieved and would avoid potentially suboptimal exposures currently being experienced by a significant number of neonate and infant patients, particularly those patients receiving doses <0.050 mg/kg. Indeed, this suggested therapeutic window is supported by data from a recently published US study, which reported a median AUC of 78 μg/l∗h in a paediatric patient population spanning all age ranges [22]. With this proposed therapeutic window, we would anticipate that TDM based dosing would be beneficial for approximately 50% (12/26) of neonate and infant patients who failed to achieve this proposed target therapeutic window at the prescribed dose levels. In comparison, this target was achieved by >90% of older patients recruited to the current study (29/31). In addition, it should be noted that one of the two older patients who failed to achieve an exposure within this proposed target window had received a reduced dose of 0.55 mg/m^2^ because of a raised bilirubin level. In the absence of using a TDM approach to treatment, the evidence provided would strongly discourage the use of doses <0.05 mg/kg in neonate and infant patients because of a high risk of the patients experiencing potentially suboptimal drug exposures. Dose reductions below this level should only be considered in cases of excessive toxicity at higher dose levels.

In summary, the present study provides clear evidence-based guidance for the dosing of vincristine in neonates and infant patients, which will positively impact the treatment of future patients in this challenging patient population. In addition, the data generated would strongly support the use of TDM as the optimal approach to treatment, as has previously been shown for carboplatin, another widely used anticancer drug in paediatric oncology [13]. The study highlights the need for further studies to expand our knowledge on drug disposition in neonate and infant patient populations.

## Authors' contributions

S.B. contributed to conceptualisation, methodology, validation, investigation, formal analysis, data curation, visualisation, and reviewing and editing. F.H. contributed to methodology, software, formal analysis, data curation, visualisation, and reviewing and editing. E.P. contributed to formal analysis, data curation, and reviewing and editing the article. G.M., D.A.T. and C.O. contributed to funding acquisition and reviewing and editing the article. G.H. contributed to methodology, validation, supervision, and reviewing and editing the article. G.J.V. contributed to conceptualisation, methodology, investigation, visualisation, writing the original article, project administration, funding acquisition and supervision.

## Role of the funding source

This work was supported in part by the National Institute for Health Research (NIHR) Research for Patient Benefit programme (PB-PG-1216-20032), 10.13039/501100000289Cancer Research UK (C9380/A25138) and the Experimental Cancer Medicine Centre Network (C9380/A25169). The views expressed are those of the authors and not necessarily those of the NIHR or the Department of Health and Social Care. None of the funding bodies played a role in the study design, the collection, analysis or interpretation of data, the writing of the report or the decision to submit the article for publication.

## Conflict of interest statement

The authors have no financial relationships relevant to the work contained in this article to disclose and no other conflicts of interest to disclose.
